# Prediabetes blunts *DPP4* genetic control of postprandial glycaemia and insulin secretion

**DOI:** 10.1007/s00125-021-05638-6

**Published:** 2022-02-22

**Authors:** Rita S. Patarrão, Nádia Duarte, Inês Coelho, Joey Ward, Rogério T. Ribeiro, Maria João Meneses, Rita Andrade, João Costa, Isabel Correia, José Manuel Boavida, Rui Duarte, Luís Gardete-Correia, José Luís Medina, Jill Pell, John Petrie, João F. Raposo, Maria Paula Macedo, Carlos Penha-Gonçalves

**Affiliations:** 1 Centro de Estudos de Doenças Crónicas (CEDOC), NOVA Medical School, Faculdade de Ciências Médicas, Universidade Nova de Lisboa, Lisbon, Portugal; 2 Instituto Gulbenkian de Ciência, Oeiras, Portugal; 3 Institute of Health & Wellbeing, University of Glasgow, Glasgow, UK; 4 Associação Protectora dos Diabéticos de Portugal/Diabetes Portugal Education and Research Center (APDP-ERC), Lisbon, Portugal; 5 Departamento de Ciências Médicas, Instituto de Biomedicina (iBiMED), Universidade de Aveiro, Aveiro, Portugal; 6 Sociedade Portuguesa de Diabetologia, Lisbon, Portugal; 7 Institute of Cardiovascular and Medical Sciences, BHF Glasgow Cardiovascular Research Centre, University of Glasgow, Glasgow, UK

**Keywords:** CD26/DPP4, Dysglycaemia, Genetic association, Hyperenergetic diet, Hyperinsulinaemia, Insulin secretion, Postprandial glucose, Prediabetes

## Abstract

**Aims/hypothesis:**

Imbalances in glucose metabolism are hallmarks of clinically silent prediabetes (defined as impaired fasting glucose and/or impaired glucose tolerance) representing dysmetabolism trajectories leading to type 2 diabetes. CD26/dipeptidyl peptidase 4 (DPP4) is a clinically proven molecular target of diabetes-controlling drugs but the *DPP4* gene control of dysglycaemia is not proven.

**Methods:**

We dissected the genetic control of post-OGTT and insulin release responses by the *DPP4* gene in a Portuguese population-based cohort of mainly European ancestry that comprised individuals with normoglycaemia and prediabetes, and in mouse experimental models of *Dpp4* deficiency and hyperenergetic diet.

**Results:**

In individuals with normoglycaemia, *DPP4* single-nucleotide variants governed glycaemic excursions (rs4664446, *p*=1.63x10^−7^) and C-peptide release responses (rs2300757, *p*=6.86x10^−5^) upon OGTT. Association with blood glucose levels was stronger at 30 min OGTT, but a higher association with the genetic control of insulin secretion was detected in later phases of the post-OGTT response, suggesting that the *DPP4* gene directly senses glucose challenges. Accordingly, in mice fed a normal chow diet but not a high-fat diet, we found that, under OGTT, expression of *Dpp4* is strongly downregulated at 30 min in the mouse liver. Strikingly, no genetic association was found in prediabetic individuals, indicating that post-OGTT control by *DPP4* is abrogated in prediabetes. Furthermore, *Dpp4* KO mice provided concordant evidence that *Dpp4* modulates post-OGTT C-peptide release in normoglycaemic but not dysmetabolic states.

**Conclusions/interpretation:**

These results showed the *DPP4* gene as a strong determinant of post-OGTT levels via glucose-sensing mechanisms that are abrogated in prediabetes. We propose that impairments in *DPP4* control of post-OGTT insulin responses are part of molecular mechanisms underlying early metabolic disturbances associated with type 2 diabetes.

**Graphical abstract:**

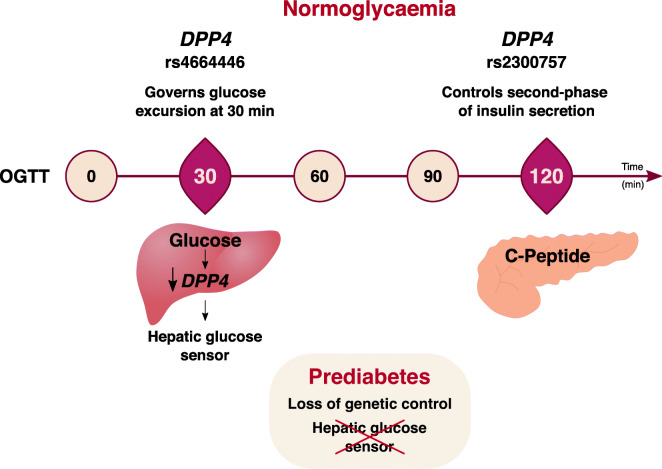

**Supplementary Information:**

The online version of this article 10.1007/s00125-021-05638-6 contains peer-reviewed but unedited supplementary material.

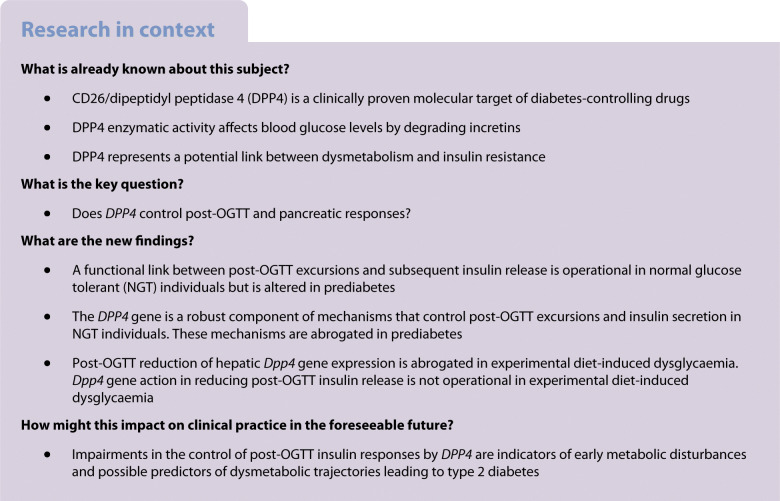



## Introduction

Responses to blood glucose variations are effected by the concurrent action of distinct mechanisms that maintain blood glucose levels homeostasis in healthy individuals [1–3]. Inefficient glycaemia homeostasis underlies clinically silent dysmetabolic states representing early stages of development of type 2 diabetes. Cross-sectional studies consistently find that prediabetes (defined as impaired fasting glucose and/or impaired glucose tolerance) has a high prevalence among apparently healthy individuals, and it is estimated that more than 470 million people will be prediabetic by 2030 [4, 5]. Prediabetes is a heterogeneous condition that encompasses a diverse set of dysglycaemic trajectories, and therefore is a poor predictor of overt type 2 diabetes development [5, 6]. Similarly to type 2 diabetes, the aetiopathogenesis of prediabetes is multifactorial and fuelled by interactions between genetic and environmental factors.

The genetic basis of type 2 diabetes susceptibility has been intensively investigated, and genome-wide association studies using multicentric approaches have identified over 120 distinct genetic loci as type 2 diabetes risk factors [7, 8]. Nevertheless, these genetic variants only account for ~20% of type 2 diabetes heritability, possibly owing to genetic heterogeneity and interactions with environmental factors [8, 9]. This implies that diverse pathogenesis and genetic mechanisms intervene in the course of type 2 diabetes natural history [9], making it difficult to pinpoint early pathogenesis factors contributing to overt type 2 diabetes. In this scenario, genetic analysis of pre-disease states offers an opportunity to identify genetic modifiers of glycaemic homeostatic control and to unveil mechanisms of glucose metabolism dysregulation preceding development of type 2 diabetes. It has been shown that genotypes predisposing to prediabetes are associated with beta-cell function and insulin secretion [10]. Accordingly, some genes were found to be associated both with diabetes and glucose impairment traits in healthy individuals [9]. This illustrates that investigating genetic mechanisms that control insulin and glucose metabolism in normoglycaemic individuals is instrumental to elucidating early metabolic impairments in diabetogenesis [9–11].

Dipeptidyl peptidase 4 (CD26/DPP4) is a ubiquitous glycoprotein occurring in two isoforms, a cell membrane-bound protein expressed on the surface of many cell types and a soluble form found in most body fluids [12, 13]. DPP4 exhibits enzymatic activity targeting a variety of substrates, but most prominently two incretin hormones: glucagon-like peptide-1 (GLP-1) and glucose-dependent insulinotropic polypeptide (GIP, also known as gastric inhibitory polypeptide). GLP-1 and GIP are gut hormones that are released in response to digestion and absorption of food in the small intestine and increase insulin secretion upon oral glucose ingestion, a phenomenon termed the 'incretin effect'. The incretin-degrading activity of DPP4 represents a potential link between dysmetabolism and insulin resistance [14]. The degradation of incretins by DPP4 decreases their insulinotropic effect and leads to higher blood glucose levels. Serum levels and activity of DPP4 are altered in many pathophysiological conditions, namely in obesity and diabetes [14–18]. It has been shown that the incretin effect is reduced in patients with type 2 diabetes, which is believed to contribute to the impairments in glucose tolerance [19, 20]. Accordingly, inhibition of DPP4 is used in clinical practice to prolong incretin action and improve glycaemic control [21]. GLP-1 levels are reduced in prediabetes [22, 23]. This indicates that the postprandial glucose/insulin axis is impaired in early dysmetabolic stages, but the involvement of DPP4 is yet to be determined.

Genetic ablation of *Dpp4* in mice [24] improves insulin sensitivity and liver glucose metabolism, and DPP4 pharmacological inhibition reduces the development of hepatic steatosis and insulin resistance in mouse models of obesity and diabetes [25, 26]. Additionally, diet-induced obesity in mice was associated with increased expression and release of hepatic DPP4, with early insulin resistance and development of hepatic steatosis [27].

It has been shown that increased glucose levels downregulate DPP4 expression in several cellular systems [28, 29], suggesting a glucose-sensing mechanism that potentially limits DPP4 anti-incretin activity, with subsequent blood glucose-lowering effects. Thus, DPP4 plays intricate physiological roles in glycaemic control but it remains unknown whether it is a relevant modulator of glucose and/or insulin metabolism during dysmetabolism.

We address this question by investigating the genetic control of *DPP4* glycaemic responses and insulin secretion post-OGTT in normoglycaemic and prediabetic individuals and in mouse models of diet-induced dysglycaemia. We postulate that association of particular *DPP4* genetic variants with post-OGTT and/or insulin regulation would provide evidence that modulation of *DPP4* gene expression has a functional role in glucose metabolism. We found significant genetic association between *DPP4* single-nucleotide variants and both increased glucose excursions and increased C-peptide release after post-OGTT in normoglycaemic but not prediabetic individuals, indicating that DPP4-dependent regulation of post-OGTT glycaemia is abrogated in early metabolic dysregulation. Surprisingly, our results show that *DPP4* regulation of blood glucose levels excursions precedes the regulation of C-peptide release, and possibly occurs through down-modulation of hepatic *DPP4* gene expression.

## Methods

### Ethics statement

All participants were volunteers and provided written informed consent for participation in this study. Ethical permits were obtained from the Ethics Committee of Associação Protectora dos Diabéticos de Portugal (APDP) and the Instituto Gulbenkian de Ciência. The study protocol adhered to the Declaration of Helsinki and was approved by the Autoridade Nacional de Protecção de Dados (3228/2013). All procedures involving mice were in accordance with national regulations (Portaria_1005/92) and European regulations (European Directive_86/609/CEE) and were approved by the Instituto Gulbenkian de Ciência Ethics Committee and by the national authority for animal welfare (Direção-Geral da Alimentação e Veterinária [DGAV]).

### Participants

The study population comprises the participants of a diabetes prevalence study performed in Portugal (PREVADIAB-2). PREVADIAB-1 recruited 5167 participants, mostly of European ancestry, attending the national healthcare system across the country who were screened for diabetes status between 2008 and 2009 [30]. In 2014, we randomly selected from within this cohort 1084 non-diabetic individuals attending 55 health units geographically spread through only continental Portugal. For all participants, a letter was sent containing information for study participation. Then, medical history was assessed, BMI was recorded and routine blood tests were performed.

### Inclusion criteria

Participants underwent a 75 g OGTT. Venous blood samples (12 h fasting) were drawn at baseline, and at 30 and 120 min after the OGTT. The diabetes status of each participant was determined using the WHO criteria for diabetes [31]. Participants fulfilling the criteria for diabetes (fasting glucose ≥7 mmol/l, 2 h post-OGTT ≥11.1 mmol/l) were excluded. Participants fulfilling the criteria for impaired fasting glucose (fasting glucose 6.1–7 mmol/l) and/or impaired glucose tolerance (2 h post-OGTT 7.8–11.1 mmol/l) were classified as prediabetic individuals (*n* = 233). The remaining participants were classified as being normal glucose tolerant (NGT, *n* = 736).

Supplemental analysis of UK Biobank data included participants meeting the following criteria: HbA_1c_ measurement, European ancestry, genetically determined sex matching self-reported sex, absence of putative sex chromosome aneuploidy, <10% missing genetic data, not determined to have poor heterozygosity. After exclusions for pairs of related individuals with kinship coefficient >0.042 (second cousin) with valid phenotype, one person was randomly excluded.

UK Biobank obtained informed consent from all participants, and analysis was conducted under generic approval from the NHS National Research Ethics Service (13 May 2016, Ref16/NW/0274) with UK Biobank approval for application #7155.

### Biochemical variables

Plasma glucose, insulin and C-peptide levels were measured at baseline, and after 30 and 120 min of OGTT. Glucose levels were measured using a glucose analyser (Olympus AU640, Beckman Coulter, Portugal). Insulin and C-peptide levels were determined using Liaison chemiluminescence assays (DiaSorin, Italy). NGT and prediabetic individuals were compared using the Mann–Whitney test in GraphPad Prism 8, Version 8.2.1 (279) (USA). The HOMA-IR was assessed [32]. Glucose and C-peptide AUCs during the OGTT were calculated according to the trapezoid method:

$$ {\displaystyle \begin{array}{l}\mathrm{Glucose}\ \mathrm{AUC}=\frac{\mathrm{Glucose}\ 0\min +\mathrm{Glucose}\ 30\min }{2}\times 30+\frac{\mathrm{Glucose}\ 30\min +\mathrm{Glucose}\ 120\min }{2}\times 90\\ {}\mathrm{C}-\mathrm{peptide}\ \left(\mathrm{AUC}\ 0-120\min \right)=\frac{\mathrm{C}-\mathrm{peptide}\ 0\min +\mathrm{C}-\mathrm{peptide}\ 120\min }{2}\times 120\end{array}} $$

### Genotyping

Genomic DNA was extracted from whole blood using Chemagen magnetic beads. DNA was quantified using PicoGreen (Invitrogen, Portugal) according to the supplier instructions. A total of 38 SNPs covering the *DPP4* region in chromosome 2 (161.9–162.1 Mb) were genotyped using the Sequenom iPlex assay (San Diego, USA) and the Sequenom MassArray K2 platform at the Instituto Gulbenkian de Ciência Genomics Unit. Genotype calling was performed blinded to affection status, and its quality was controlled using two samples with known genotypes. SNPs deviating from Hardy–Weinberg equilibrium were excluded (*p*<0.05). The final dataset comprised 969 participants with a non-missing genotyping rate >95%, genotyped for 33 SNPs that passed the exclusion criteria (minor allele frequency <8% or call rate <99%) (ESM Table 1). A linkage disequilibrium (LD) map was generated by Haploview4.2 (ESM Fig. 1a).

### Genetic association analysis

Quantitative trait association analysis was performed using the PLINK software package, and nominal *p* values for 33 *DPP4* SNPs were obtained for allelic and genotypic association under the additive model using age and BMI as covariates. Empirical *p* values were obtained using permutation tests (1x10^6^ label permutations), and conditional analysis was performed by testing association with pairwise SNP covariates. All analyses used routines implemented in PLINK. Data storage and interfacing with PLINK used BC|Gene version_3.6–036 [33].

### Mouse studies

C57BL/6-DPP4tm1Nwa/Orl (*Dpp4* KO) mice were purchased from European Mouse Mutant Archive (Infrafrontier, Germany, EM:02577; www.infrafrontier.eu/search?keyword=02577). C57BL/6 (C57BL/6J, JAX mouse strain, were purchased from Charles River Laboratories Europe (www.criver.com/products-services/find-model/jax-c57bl6j-mice?region=3616) and bred in-house) and *Dpp4* KO mice were bred and housed under a 12 h light/dark cycle in specific pathogen-free housing facilities at the Instituto Gulbenkian de Ciência. For hyperenergetic regimen experiments, C57BL/6 and *Dpp4* KO female mice at 5–6 weeks of age were maintained with free access to water and regular chow (Chow-RM3A, UK) or hyperenergetic (hypercaloric) diet (HCD, TD.88137, Harlan, USA, with 42% energy from fat [61.8% from saturated fatty acids] and 42.7% energy from carbohydrates [63% from sugar]) for 6 weeks. OGTT was performed after overnight fasting. Chow- or HCD-fed mice were gavaged with glucose (1.5 g/kg body weight), and blood samples were collected at baseline and at 15, 30, 60 and 120 min post-gavage. C-peptide was quantified using a mouse C-peptide ELISA kit (Crystal Chem, USA). C-peptide AUC was calculated as described above.

For gene expression analysis, C57BL/6 mice were fasted overnight, and, 30 min after gavage with glucose (1.5 g/kg of body weight; glucose group) or water (control group), the liver and ~2 cm of duodenum, jejunum and ileum were collected. C57BL/6 mice were maintained on HCD for 12 weeks, OGTT was performed, and gene expression was analysed as described above. RNA was extracted using RNeasy Mini Kit (Qiagen, Germany) according to the manufacturer instructions, converted to cDNA (First Strand cDNA Synthesis Kit, Roche, Portugal) and amplified using *Dpp4* (Mm00494538_m1) Taqman Gene expression assays (Applied Biosystems, USA). Mouse endogenous *Gapdh* control (Applied Biosystems, Portugal) was used in multiplex PCR with the target gene. PCR reactions were performed using the ABI QuantStudio-384 (Applied Biosystems, Portugal). Relative quantification was calculated by the ${2}^{-\Delta \Delta {\mathrm{C}}_{\mathrm{t}}}$ method, and expression values were normalised to the mean value of the control group in the liver and to the duodenum control mean value for the small intestine. Blood glucose levels were measured before and 30 min after gavage.

## Results

### Glucose and C-peptide post-OGTT responses in normoglycaemic and prediabetic individuals

We evaluated post-OGTT metabolism in PREVADIAB-2 individuals by analysing blood glucose levels, insulinaemia and C-peptide levels at baseline, and 30 and 120 min after OGTT. Fasting blood glucose levels and at 120 min revealed that 24% of the 969 non-diabetic individuals fulfilled the WHO criteria for prediabetes. As expected, fasting insulinaemia, insulin resistance (HOMA-IR) and BMI were higher in prediabetic individuals compared with NGT individuals (Table 1).

**Table 1:** Glucose metabolism variables of NGT and prediabetic participants in the PREVADIAB-2 cohort

Variable	NGT	Prediabetes
Participants (*n*)	736	233
Sex, M/F (%)	292/444 (39.7/60.3)	93/140 (39.9/60.1)
Age (years)	58.5 ± 13.3	65.0 ± 10.5***
BMI (kg/m^2^)	26.8 ± 4.2	28.8 ± 4.2***
HOMA-IR	1.55 ± 1.01	2.34 ± 1.56***
Fasting insulin (pmol/l)	48.9 ± 29.7	65.9 ± 41.7***
Fasting glucose (mmol/l)	4.90 ± 0.49	5.49 ± 0.70***
30 min OGTT glucose (mmol/l)	8.02 ± 1.55	9.63 ± 1.57***
120 min OGTT glucose (mmol/l)	5.45 ± 1.20	8.29 ± 1.42***
Glucose AUC (mmol/l × min)	800.1 ± 118.2	1033.0 ± 108.2***
Fasting C-peptide (nmol/l)	0.69 ± 0.27	0.85 ± 0.34***
30 min OGTT C-peptide (nmol/l)	2.25 ± 0.84	2.19 ± 0.98
120 min OGTT C-peptide (nmol/l)	2.50 ± 1.13	3.71 ± 1.41***
C-peptide AUC (0–120) (nmol/l × min)	191.5 ± 78.8	273.7 ± 98.3***

Values are mean ± SD

***p<0.001 vs NGT (Mann–Whitney test)

**Fig. 1:**
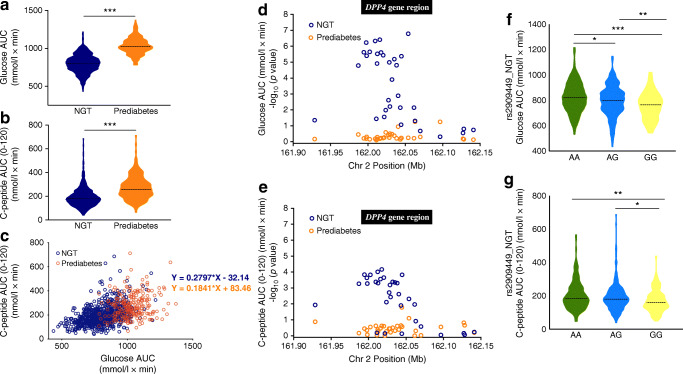
*DPP4* genetic association with post-OGTT plasma glucose and C-peptide levels in NGT and prediabetic participants. (**a**, **b**) The AUC of the glucose excursion (**a**) and the C-peptide response AUC (0–120 min) (**b**) were calculated for each participant. The violin plots represent the probability density, the median, the IQR and the 95% CI of the phenotype distributions in 736 NGT (blue) and 233 prediabetic (orange) participants. ***p<0.001, unpaired *t* test (Mann–Whitney). (**c**) Correlation of plasma glucose AUC and C-peptide AUC (0–120 min) during OGTT, in NGT participants (blue circles; Pearson’s correlation, *r*^2^=0.193, *p*<0.001) and in prediabetic participants (orange circles; Pearson’s correlation, *r*^2^=0.055, *p*=0.0002). (**d**, **e**) Plots of quantitative trait locus analysis for 33 SNPs in the *DPP4* gene region, testing for association with (**d**) plasma levels of glucose AUC and (**e**) C-peptide AUC (0–120 min) for NGT participants (blue circles) and prediabetic participants (orange circles). Results represent the nominal –log10 (*p* value) for allelic association and the SNP position in chromosome 2 is represented in Mb and a bar representing the *DPP4* gene region is shown above the plots. (**f**, **g**) Violin plots of rs2909449 genotypic effects on plasma glucose AUC (**f**) and C-peptide AUC (0–120 min) (**g**) during OGTT in NGT participants (green, ancestral allele homozygotes; blue, heterozygotes; yellow, minor allele homozygotes). *p<0.05, **p<0.01 and ***p<0.001 by Kruskal–Wallis test with Dunn’s correction for multiple comparisons. The plots represent the probability density, the median, the IQR and the 95% CI of the phenotypic distributions per genotype class totalling 736 NGT participants. Chr, chromosome

Analysis of blood glucose levels during OGTT evaluated both by point-wise analysis or AUC (Table 1, Fig. 1a) confirmed the expected increase of glycaemic excursions in prediabetic individuals compared with NGT individuals [34]. Consequently, the C-peptide response at 120 min post-OGTT was clearly higher in prediabetic individuals than in NGT individuals (Table 1, Fig. 1b), showing that post-OGTT pancreatic insulin release is augmented in prediabetes. Our results show that the glucose AUC has a higher correlation with C-peptide (AUC 0-120 min) in normoglycaemia than in prediabetes (Fig. 1c), indicating more efficient beta-cell function.

### *DPP4* gene variants control glucose responses in normoglycaemic individuals

We investigated whether the gene coding for *DPP4* was involved in the post-OGTT response. Non-diabetic participants of the PREVADIAB-2 cohort (*n* = 969) were genotyped for 33 SNPs covering a genomic region of approximately 212 kb encompassing the *DPP4* gene (ESM Table 1). A large LD block was identified, spanning intron 3 to 23 (ESM Fig. 1a). The role of *DPP4* genetic variance in controlling glucose metabolism was separately evaluated in NGT and prediabetic individuals.

Quantitative trait locus analysis in NGT individuals using allelic (Fig. 1d) and additive models (ESM Table 2) revealed a highly significant association with glucose AUC. Strong nominal association with glucose AUC was found across the *DPP4* gene region that spans intron 3 to 23. The highest association was found with rs4664446 in intron 2 (*p*=1.6x10^−7^, allelic model; *p*=1.17x10^−6^, additive model), mapping outside the large LD block in *DPP4* gene. This genetic association was lost in individuals with prediabetes (ESM Fig. 2a).

Three additional peaks of association with glucose AUC were found within the LD region at rs2909449 in intron 20 (*p*=1.96x10^−6^, additive model), rs2268890 in intron 18 (*p*=2.27x10^−6^, additive model) and rs6432708 in intron 8 (*p*=2.87x10^−5^, additive model) (Fig. 1d, ESM Fig. 2, ESM Table 2). Covariate analysis suggests that these association signals within the LD region are unlinked to the rs4664446 signal (ESM Fig. 1b).

### *DPP4* gene variants control C-peptide responses in normoglycaemic individuals

Quantitative trait locus analysis in NGT individuals under allelic and additive models also showed an association profile of *DPP4* gene with C-peptide levels after OGTT (AUC 0–120 min), albeit with lower significance levels compared with blood glucose levels (Fig. 1e and g, ESM Table 3). The strongest association signal at rs16822665 (*p*=4.87x10^−4^) is probably unlinked to other associated SNPs, namely rs7565794, rs1014444 and rs2300757, as determined by conditional analysis (ESM Fig. 1b). This genetic association was lost in prediabetes (ESM Fig. 2b). The statistical significance of these results resisted permutation tests (ESM Tables 2 and 3), indicating that the *DPP4* gene is a robust component of mechanisms that control post-OGTT excursion and insulin secretion in normoglycaemic individuals.

We further explored the involvement of the *DPP4* gene in glycaemic responses by using HbA_1c_ as a proxy for imbalanced dysglycaemia in the UK Biobank cohort. We found that four SNPs (rs7565794, rs1014444, rs2300757 and rs16822665) with highest association with increased C-peptide (AUC_0–120 min) in the PREVADIAB-2 cohort also showed association signals with increased HbA_1c_ levels in the UK Biobank cohort (ESM Table 4). This result strongly suggests that our report on association of glycaemic response traits with *DPP4* is not specific to the genetic structure of the PREVADIAB-2 cohort.

### *DPP4* acts a glucose sensor in post-OGTT responses

Point-wise analysis of blood glucose levels responses revealed that association signals in NGT individuals were generally more robust at 30 min OGTT as compared with 120 min OGTT, and non-significant at fasting (Table 2, ESM Table 5). This strongly suggests that the *DPP4* gene exerts control of post-OGTT excursions in an early time frame. In contrast, control of C-peptide levels by *DPP4* gene variants was not apparent at 30 min OGTT but was stronger at 120 min (Table 2, ESM Table 6). Interestingly, peaks of association with glycaemia at 30 min were in close proximity to the stronger association signals with C-peptide levels at 120 min, raising the possibility that control of blood glucose levels by *DPP4* gene variants indirectly affects subsequent C-peptide secretion responses. Accordingly, analysis of the SNPs with highest association phenotypic effects by genotype class revealed that alleles conferring lower glucose excursions in NGT were also associated with lower C-peptide responses, as shown for rs2909449 in Fig. 1f and g. Together, these observations suggest that the *DPP4* gene region senses post-OGTT absorption and directly controls glucose excursions with indirect effects on insulin secretion.

**Table 2:** *DPP4* association peaks with plasma glucose and C-peptide levels at baseline and 30 and 120 min after OGTT in NGT individuals

SNP ID		0 min		30 min		120 min	
	Minor allele	β	*p*_*asymp*_	β	*p*_*asymp*_	β	*p*_*asymp*_
Glucose							
rs2909449	G	−0.75	1.02 × 10^−1^	−5.75	7.47 × 10^−5^	−3.38	2.84 × 10^−3^
rs2268890	A	−0.68	1.37 × 10^−1^	−5.63	1.04 × 10^−4^	−3.49	2.06 × 10^−3^
rs6432708	C	−0.58	2.17 × 10^−1^	−4.98	7.69 × 10^−4^	−3.28	4.44 × 10^−3^
rs4664446	G	−1.48	1.10 × 10^−3^	−6.10	1.89 × 10^−5^	−2.62	1.91 × 10^−2^
C-peptide							
rs7565794	C	0.11	1.02 × 10^−2^	0.01	9.16 × 10^−1^	0.66	4.04 × 10^−4^
rs1014444	G	0.12	4.25 × 10^−3^	0.08	5.54 × 10^−1^	0.66	2.86 × 10^−4^
rs2300757	G	0.12	5.40 × 10^−3^	0.03	8.26 × 10^−1^	0.68	2.91 × 10^−4^
rs16822665	T	0.12	7.71 × 10^−3^	0.08	5.69 × 10^−1^	0.65	7.03 × 10^−4^

β, regression coefficient; *p*_*asymp*_*,* asymptotic *p* value for linear regression under the additive model adjusted for age and BMI

**Fig. 2:**
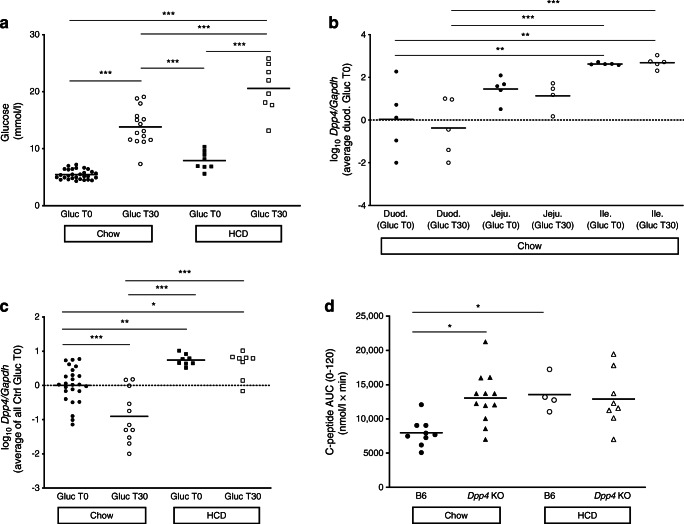
*Dpp4* is implicated in mouse experimental post-OGTT responses under normal diet or HCD. (**a**) Blood glucose levels before and at 30 min (Gluc T30) after gavage with 1.5g/kg glucose (Gluc). (**b**, **c**) mRNA expression of the mouse *Dpp4* gene at T30 after gavage with 1.5g/kg glucose (Gluc T30) or with water was quantified in portions of the duodenum (Duod.), jejunum (Jeju.) and ileum (Ile.) (**b**) and the liver (**c**) by real-time PCR. Expression levels were normalised to those of the mouse endogenous control *Gapdh* and the mean value for duodenum (**b**) or liver (**c**) from control mice gavaged with water. Values are represented on a logarithmic scale, *n*=5–25 mice per group. (**d**) Serum C-peptide levels were measured by ELISA at baseline (0) and 15, 30, 60 and 120 min after OGTT in C57BL/6 (B6) and *Dpp4* KO mice on regular chow or after 6 weeks of HCD. The plot represents the AUC (0–120 min), *n*=4–12 mice per group. *p<0.05, **p<0.01 and ***p<0.001 (one-way ANOVA using Tukey’s correction)

To experimentally test whether *Dpp4* gene expression is controlled by post-OGTT sensing, we analysed mouse *Dpp4* RNA levels 30 min after oral glucose challenge. Blood glucose levels and *Dpp4* gene expression in liver and small intestine were analysed (Fig. 2b and c). We observed the expected rise in blood glucose levels at 30 min in chow-fed mice (Fig. 2a). As reported previously, expression of *Dpp4* in the small intestine was particularly high in the ileum [35] but was not significantly altered with glucose ingestion in other intestine regions (Fig. 2b). *Dpp4* expression was significantly downregulated in the liver of chow-fed mice that received glucose (Fig. 2c). These results indicate that the liver responds to glucose challenge by decreasing expression of *Dpp4* [36], raising the possibility that *DPP4* gene expression in normoglycaemic individuals is under the control of a hepatic glucose sensor, resulting in reduced post-OGTT excursions.

### Prediabetes abrogates *DPP4* genetic control of glucose and C-peptide responses

In contrast with normoglycaemic individuals, no significant association of glucose AUC or C-peptide AUC (0–120 min) was found with the tested *DPP4* SNPs in prediabetes (Fig. 1d and e, ESM Fig. 2a and 2b, ESM Tables 2 and 3). Similarly, point-wise analysis of post-OGTT and C-peptide levels did not yield significant results (data not shown). These results indicate that the control of post-OGTT mediated by the *DPP4* gene is abolished in prediabetes, and is possibly implicated in early manifestations of dysglycaemia.

We used a mouse model under HCD to mimic prediabetes and determine whether hyperglycaemia affects *Dpp4* expression (Fig. 2a and c). The glucose excursion 30 min after OGTT was significantly higher in HCD-fed mice (Fig. 2a). In addition, hepatic *Dpp4* expression was increased compared with chow-fed mice (Fig. 2c), as previously described [27]. Surprisingly, the glucose challenge failed to significantly reduce liver *Dpp4* expression, as observed in normoglycaemic mice, suggesting that the hepatic sensing of oral glucose is abrogated in hyperglycaemia (Fig. 2c).

We further tested whether *Dpp4* effects on post-OGTT insulin secretion were impaired in dysmetabolic conditions using a mouse *Dpp4* gene knockout model. *Dpp4* ablation resulted in an increased insulin secretion response during the OGTT in chow-fed mice, confirming that *Dpp4* acts to reduce post-OGTT insulin response to glucose challenge under standard metabolic conditions. Notably, this differential response was blunted in HCD-fed mice, as *Dpp4* KO mice and wild-type animals showed similar increases in post-OGTT insulin secretion (Fig. 2d). This finding corroborates our data indicating that the genetic effect of *DPP4* on post-OGTT/insulin axis responses is abrogated in early dysmetabolic stages.

## Discussion

This study revealed that the *DPP4* gene controls responses to post-OGTT challenges in normoglycaemic individuals. Specifically, we found that genetic polymorphisms in the *DPP4* locus, spanning intron 3 to 23, control both glucose excursions and C-peptide release after glucose ingestion in normoglycaemia but not in prediabetic individuals. Notably, genetic control of glucose excursions was more pronounced at early time points in the OGTT, and the genetic effects on insulin secretion were stronger in later phases. Together, the results obtained from human participants and experimental models support the proposal of a post-OGTT sensing genetic mechanism for fine-tuning the response to glucose ingestion that operates via *DPP4* and is overridden in dysglycaemia.

Importantly, we found that *DPP4* alleles acting to reduced post-OGTT excursions were also associated with reduced C-peptide release in NGT, suggesting that the genetic effect on the pancreatic response was subsequent to the control over the glucose excursions (Fig. 1f and g; Table 2). In agreement, association of *DPP4* SNPs with post-OGTT C-peptide release was not detected at 30 min but only at 120 min after glucose challenge. On the other hand, association with blood glucose levels were readily detected at 30 min (Table 2), again suggesting that the genetic control of glucose excursions conditioned the pancreatic response. This explains the detection of stronger genetic association signals with glucose excursions (glucose AUC 0–120 min) compared with pancreatic responses (C-peptide AUC 0–120 min). These findings show that, in normoglycaemia, *DPP4* gene responds to glucose ingestion and indirectly regulates its excursion in blood, thus forming part of a post-OGTT sensing mechanism that may govern the subsequent C-peptide release response. In addition, we have not found significant association of *DPP4* SNPs with other metabolic traits related to lipid metabolism. Likewise, no association was found with biochemical measurements related to liver function (aspartate aminotransferase, alanine aminotransferase and γ-glutamyl transferase) or insulin resistance (HOMA-IR).

Genotypes at the *DPP4* locus had no effect on controlling serum DPP4 enzymatic levels (data not shown), suggesting that the action *DPP4* SNPs in controlling post-OGTT and insulin secretion is not detectable by assessing DPP4 levels in peripheral blood. This reinforces the notion that the observed genetic control over blood glucose levels and C-peptide secretion is not explained by direct regulation of the incretin effect.

Several mechanisms have been suggested to be involved in the glucose-lowering effect of DPP4 inhibitors in rodents and humans [21] that do not affect levels of plasma DPP4 activity. In mice, DPP4 inhibition in the gut but not in the plasma was achieved by low doses of DPP4 inhibitors, leading to glucose regulation through local effects on GLP-1 inactivation [37]. DPP4 inhibitors were shown to prevent inactivation of GLP-1 in the gut or in pancreatic islets, contributing to reduced hepatic glucose production or increased insulin secretion and decreased glucagon secretion [38]. These data suggest that DPP4 controls glycaemic responses using mechanisms that do not affect DPP4 plasma levels.

Reduction of DPP4 expression upon exposure to high glucose was reported to occur in vitro in an epithelium intestinal cell line [29] and adipocytes [28]. We found that liver expression of DPP4 mRNA was downregulated upon in vivo glucose exposure. These results suggest that glucose-induced modulation of DPP4 expression occurs in the liver and may have an indirect effect on hepatic functions through glucose uptake/release, contributing to regulation of glucose excursions. In parallel, exogenous GLP-1 was shown to suppress hepatic glucose output in humans independently of plasma insulin, C-peptide levels and without alterations in NEFA levels [36]. It has been proposed that the direct hepatic effects of GLP-1 on glucose output may be mediated by biological active degradation products of GLP-1, independent of canonical GLP-1 receptor (GLP-1r) [39]. Together, this evidence raises the possibility that the *DPP4* gene senses and responds to post-OGTT through down-regulation of RNA expression in the liver but not in the gut, possibly decreasing liver glucose output and leading to glycaemic-lowering effects. The precise glucose-sensing mechanisms that determine differential *DPP4* gene regulation in the liver and its effects on GLP-1 liver clearance warrant further research.

Several studies have suggested an alternative model of post-OGTT GLP-1 action through a neural circuit originating in the hepatic portal area [40]. Evidence of rapid degradation of GLP-1 in the hepatoportal bed even in the presence of vildagliptin (a DPP4 inhibitor) suggests that a mechanism other than endocrine action accounts for the glucose-lowering effect of DPP4 inhibition [41]. Infusion of GLP-1 and glucose intraportally caused an increase in glucose-stimulated insulin secretion that was attenuated by neural blockade. GLP-1r is expressed by vagal afferent neurons that innervate the abdominal organs including the hepatoportal region [40, 41]. It was observed that selectively blocking GLP-1r in the portal circulation caused significant impairment in glucose tolerance. These findings suggest that vagal GLP-1r neurons innervating the hepatic portal region mediate the glucose-lowering effect of endogenous GLP-1, and that local blockade of portal GLP-1r causes glucose intolerance.

The striking finding that glucose excursions or C-peptide responses in prediabetic individuals are not controlled by *DPP4* gene variants suggests that dysglycaemic states abrogate post-OGTT sensing by *DPP4*. A study using a cohort of overweight individuals with 70% prediabetics [42] showed that one SNP, rs6741949, in intron 2 of the *DPP4* locus, was weakly associated (*p*=0.0447) with oral glucose-stimulated GLP-1 increase. Association of the minor allele of this SNP with increased insulin secretion was only found within high-body-fat individuals, suggesting that this association mainly depends on increased adiposity [42]. Other studies have shown that *DPP4* genetic polymorphisms in type 2 diabetes are associated with serum lipid levels, with an allele mutation of A to G in rs3788979 being associated with reduced ApoB level [43–45]. We also found no associations of *DPP4* with serum lipids in our prediabetic group that comprised individuals with impaired fasting glucose, impaired glucose tolerance or both combined. Nevertheless, it remains to be determined whether these prediabetes sub-phenotypes equally contribute to the loss of post-OGTT sensing by *DPP4*.

Analysis of C-peptide secretion upon OGTT in *Dpp4* KO mice confirmed the role of *Dpp4* in regulation of the glucose/insulin axis, which may result at least in part from an increased incretin effect. We cannot exclude the possibility that diet-induced hyperglycaemia in our mouse model alters the incretin effect. Indeed, the loss of effect of diet-induced hyperglycaemia on C-peptide secretion in *Dpp4* KO mice together with lack of *Dpp4* association with C-peptide levels in prediabetes suggests that hyperglycaemia may override the *Dpp4* genetic control independently of the incretin effect. Our finding that post-OGTT excursions in HCD-fed mice failed to reduce *Dpp4* expression reinforces the hypothesis that glucose sensing by the *DPP4* gene is impaired in dysmetabolic states. Further, this raises the possibility that epigenetic modifications imposed by exposure to sustained dysmetabolic conditions abrogate *DPP4* gene action on post-OGTT metabolism. Whether such genetic modifications are reversible in individuals with diabetic dysmetabolism should be further addressed. Current therapeutic recommendations only target overall DPP4 activity. Our animal work suggests that the lost hepatic capacity of post-OGTT sensing by the *Dpp4* gene may be a relevant therapeutic driver.

In summary, our data indicate that *DPP4* genetic polymorphisms are involved in the control of post-OGTT excursions and subsequent C-peptide release responses through glucose-sensing mechanisms that are abrogated in prediabetes.

## Supplementary information

ESM (PDF 809 kb)

## Data Availability

The datasets generated during and/or analysed during the current study are available from the corresponding author on reasonable request.

## Abbreviations

DPP4: Dipeptidyl peptidase 4
GIP: Glucose-dependent insulinotropic polypeptide
GLP-1: Glucagon-like peptide-1
GLP-1r: GLP-1 receptor
HCD: Hyperenergetic diet
LD: Linkage disequilibrium
NGT: Normal glucose tolerant
OGTT: Oral glucose tolerance test
PREVADIAB: Diabetes prevalence study in Portugal
