# Supplementary material for: Prediabetes blunts DPP4 genetic control of postprandial glycaemia and insulin secretion
Source: Diabetologia. 2022 Feb 22;65(5):861–71. doi: 10.1007/s00125-021-05638-6 (PMC8960640; doi:10.1007/s00125-021-05638-6)
Supplement: Supplementary file 1 — (PDF 809 kb) [file 125_2021_5638_MOESM1_ESM.pdf]

Patarrão et al: Prediabetes blunts *DPP4* genetic control of postprandial glycaemia and insulin secretion

Electronic Supplementary Material (ESM)

ESM Table 1 Description of 33 *DDP4* SNPs genotyped in this study.

SNP	bp	Alleles	MAF	Position in gene
rs10930036	161928813	C/T	0.33	Downstream
rs2052400	161987158	G/A	0.34	Downstream
rs12617656	161994637	T/C	0.29	Intron 25
rs2970933	161997766	G/A	0.38	Intron 23
rs2287509	161998398	T/G	0.37	Intron 23
rs7565794	162002490	T/C	0.29	Intron 23
rs2909449	162009828	A/G	0.38	Intron 20
rs2909450	162011281	G/A	0.28	Intron 19
rs1014444	162012348	A/G	0.31	Intron 19
rs1014445	162012693	G/A	0.35	Intron 19
rs2268890	162015982	G/A	0.37	Intron 18
rs2300757	162017660	G/C	0.28	Intron 16
rs12692646	162020518	T/A	0.28	Intron 13
rs138687963	162021895	A/-	0.28	Intron 11
rs13409045	162023873	T/C	0.36	Intron 10
rs2300755	162027452	C/T	0.33	Intron 10
rs2268889	162028600	G/A	0.30	Intron 10
rs4664443	162029379	A/G	0.36	Intron 10
rs2284870	162032217	A/G	0.32	Intron 10
rs6432708	162035338	T/C	0.35	Intron 8
rs10930040	162038256	G/A	0.28	Intron 8
rs71408196	162040386	-/AT	0.11	Intron 5
rs12995983	162043293	T/C	0.26	Intron 5
rs3788979	162044379	G/A	0.09	Intron 5
rs16822665	162044817	C/T	0.28	Intron 5
rs4664446	162053893	A/G	0.44	Intron 2
rs873826	162061561	G/A	0.32	Intron 2
rs741529	162062979	G/A	0.14	Intron 2
rs35280626	162070034	C/T	0.27	Intron 2
rs4436979	162096640	T/C	0.49	Upstream
rs2052352	162127239	C/T	0.42	Upstream
rs13431489	162128319	A/G	0.39	Upstream
rs1990761	162141193	A/G	0.30	Upstream

bp (position in chromosome 2 according to GRCh38.p10); Alleles (ancestral/minor); MAF (minor allele frequency in PREVADIAB-2 cohort); Relative position in CD26 exon-intron structure. rs138687963 and rs71408196 are indels.

ESM Table 2 Quantitative trait locus analysis of Glucose AUC during the OGTT with SNPs in the *DPP4* gene region.

		NGT (n=736)				Prediabetes (n=233)		
**SNP**	**A**	**BETA**	**SE**	**P_{asymp}**	**$P_{emp.}$**	**BETA**	**SE**	**$P_{asymp.}$**
rs10930036	T	-186.20	113.20	1.01E-01	1.01E-01	-45.67	212.50	8.30E-01
rs2052400	A	-433.00	106.70	5.48E-05	4.90E-05	-117.80	207.80	5.71E-01
rs12617656	C	460.60	114.80	6.69E-05	5.70E-05	8.06	213.90	9.70E-01
rs2970933	A	-494.70	106.50	4.10E-06	4.00E-06	-27.87	197.90	8.88E-01
rs2287509	G	-461.20	108.00	2.23E-05	1.70E-05	-97.08	197.50	6.24E-01
rs7565794	C	463.10	113.90	5.34E-05	5.40E-05	-15.99	212.00	9.40E-01
**rs2909449**	**G**	**-509.00**	**106.10**	**1.96E-06**	**3.00E-06**	**-12.22**	**194.60**	**9.50E-01**
rs2909450	A	281.00	119.30	1.87E-02	1.89E-02	-51.02	209.10	8.08E-01
rs1014444	G	429.00	111.40	1.29E-04	1.24E-04	10.81	206.30	9.58E-01
rs1014445	A	-465.10	107.60	1.75E-05	1.90E-05	-57.15	208.00	7.84E-01
**rs2268890**	**A**	**-505.50**	**106.00**	**2.27E-06**	**3.00E-06**	**-23.69**	**197.40**	**9.05E-01**
rs2300757	G	457.10	115.40	8.29E-05	7.70E-05	22.10	211.90	9.17E-01
rs12692646	A	468.30	115.80	5.80E-05	5.20E-05	25.04	215.10	9.07E-01
rs138687963	D	308.30	119.20	9.91E-03	9.93E-03	-129.40	207.40	5.33E-01
rs13409045	T	209.80	106.80	5.00E-02	5.00E-02	157.00	201.50	4.37E-01
rs2300755	T	271.30	109.50	1.34E-02	1.33E-02	99.99	201.00	6.19E-01
rs22688890	A	409.90	114.60	3.73E-04	3.19E-04	46.39	204.00	8.20E-01
rs4664443	G	228.30	106.80	3.29E-02	3.29E-02	128.20	202.60	5.28E-01
rs2284870	G	346.00	112.30	2.15E-03	2.15E-03	127.60	201.60	5.27E-01
**rs6432708**	**C**	**-456.40**	**108.40**	**2.87E-05**	**3.10E-05**	**-79.69**	**209.80**	**7.04E-01**
rs10930040	G	434.50	117.00	2.21E-04	2.06E-04	15.83	215.10	9.41E-01
rs71408196	I	518.70	167.70	2.06E-03	2.07E-03	321.20	276.50	2.47E-01
rs12995983	C	-256.10	119.00	3.17E-02	3.18E-02	197.70	231.10	3.93E-01
rs3788979	A	214.50	177.90	2.28E-01	2.28E-01	-155.50	328.90	6.37E-01
rs16822665	T	457.10	117.10	1.04E-04	8.80E-05	20.71	217.20	9.24E-01
**rs4664446**	**G**	**-511.40**	**104.30**	**1.17E-06**	**3.00E-06**	**197.20**	**184.30**	**2.86E-01**
rs873826	A	-260.90	112.90	2.12E-02	2.12E-02	147.30	208.50	4.81E-01
rs741529	A	212.50	151.60	1.62E-01	1.62E-01	430.10	262.70	1.03E-01
rs35280626	T	-146.10	119.00	2.20E-01	2.20E-01	213.00	212.60	3.18E-01
rs4436979	C	-144.20	106.70	1.77E-01	1.77E-01	-312.90	199.20	1.18E-01
rs2052352	T	87.90	104.10	3.99E-01	3.99E-01	110.10	186.70	5.56E-01
rs13431489	G	146.40	106.30	1.69E-01	1.69E-01	102.60	189.20	5.88E-01
rs1990761	G	-134.60	115.20	2.43E-01	2.44E-01	126.90	209.90	5.46E-01

Abbreviations: A, minor allele; BETA, regression coefficient; SE, standard error; P_{asymp}, asymptotic P-value for linear regression under the additive model adjusted for age and BMI; P_{emp}, empirical pointwise P-value (10^6 permutations). SNP's representing peaks of association are highlighted in bold.

ESM Table 3 Quantitative trait loucs analysis of C-peptide AUC (0-120) during the OGTT with SNPs in the *DPP4* gene region.

		NGT (n=736)				Prediabetes (n=233)		
**SNP**	**A**	**BETA**	**SE**	**P*asymp***	**P*emp.***	**BETA**	**SE**	**P*asymp.***
rs10930036	T	-30.37	12.62	1.64E-02	1.65E-02	-42.59	31.17	1.73E-01
rs2052400	A	-38.41	11.98	1.41E-03	1.36E-03	-5.30	30.63	8.63E-01
rs12617656	C	45.36	12.88	4.58E-04	4.58E-04	19.47	31.49	5.37E-01
rs2970933	A	-39.34	11.99	1.09E-03	1.09E-03	-22.41	29.12	4.42E-01
rs2287509	G	-37.96	12.13	1.83E-03	1.79E-03	-7.73	29.12	7.91E-01
**rs7565794**	**C**	**46.21**	**12.78**	**3.21E-04**	**3.11E-04**	**21.45**	**31.20**	**4.92E-01**
rs2909449	G	-38.21	11.92	1.41E-03	1.38E-03	-30.21	28.60	2.92E-01
rs2909450	A	1.14	13.40	9.33E-01	9.33E-01	14.09	30.80	6.48E-01
**rs1014444**	**G**	**46.83**	**12.47**	**1.87E-04**	**1.95E-04**	**15.58**	**30.37**	**6.09E-01**
rs1014445	A	-37.96	12.08	1.74E-03	1.72E-03	-10.62	30.64	7.29E-01
rs2268890	A	-39.12	11.92	1.08E-03	1.06E-03	-26.00	29.03	3.71E-01
**rs2300757**	**G**	**48.47**	**12.97**	**2.02E-04**	**1.89E-04**	**24.01**	**31.22**	**4.43E-01**
rs12692646	A	45.54	13.02	4.98E-04	5.05E-04	27.39	31.64	3.88E-01
rs138687963	D	1.91	13.41	8.87E-01	8.87E-01	5.54	30.58	8.56E-01
rs13409045	T	30.87	11.91	9.78E-03	9.76E-03	4.37	29.73	8.83E-01
rs2300755	T	37.24	12.22	2.41E-03	2.42E-03	19.83	29.60	5.04E-01
rs2268889	A	40.22	12.85	1.83E-03	1.85E-03	20.50	30.03	4.96E-01
rs4664443	G	33.65	11.92	4.91E-03	4.92E-03	1.18	29.88	9.69E-01
rs2284870	G	41.46	12.55	1.01E-03	1.04E-03	24.84	29.67	4.03E-01
rs6432708	C	-38.65	12.16	1.54E-03	1.51E-03	-11.84	30.90	7.02E-01
rs10930040	G	42.46	13.05	1.19E-03	1.20E-03	28.80	31.63	3.64E-01
rs71408196	I	55.87	18.79	3.05E-03	3.19E-03	-55.13	40.69	1.77E-01
rs12995983	C	-35.04	13.25	8.36E-03	8.42E-03	-26.52	34.05	4.37E-01
rs3788979	A	-8.95	19.96	6.54E-01	6.53E-01	116.80	47.82	1.54E-02
**rs16822665**	**T**	**46.02**	**13.13**	**4.87E-04**	**4.99E-04**	**26.97**	**31.95**	**4.00E-01**
rs4664446	G	-29.10	11.82	1.41E-02	1.42E-02	-44.60	27.06	1.01E-01
rs873826	A	-28.85	12.58	2.21E-02	2.20E-02	16.92	30.73	5.82E-01
rs741529	A	22.38	16.99	1.88E-01	1.88E-01	-44.32	38.83	2.55E-01
rs35280626	T	-16.98	13.23	2.00E-01	2.01E-01	29.45	31.33	3.48E-01
rs4436979	C	-7.70	11.94	5.19E-01	5.20E-01	34.97	29.42	2.36E-01
rs2052352	T	10.84	11.65	3.52E-01	3.53E-01	-43.58	27.36	1.13E-01
rs13431489	G	11.65	11.89	3.28E-01	3.28E-01	-40.52	27.75	1.46E-01
rs1990761	G	-9.32	12.88	4.69E-01	4.70E-01	-1.65	30.94	9.58E-01

Abbreviations: A, minor allele; BETA, regression coefficient; SE, standard error; P*asymp,* asymptotic P-value for linear regression under the additive model adjusted for age and BMI; P*emp,* empirical pointwise P-value (10^6 permutations). SNP's representing peaks of association are highlighted in bold.

ESM Table 4 Quantitative trait locus analysis of HbA1c levels in the UK Biobank dataset with OGTT with SNPs in the *DPP4* gene region.

SNP	BETA	SE	P	N	P.FDR
rs10930036	-0.0565	0.0166	6.74E-04	325225	4.42E-02
rs2052400	-0.0233	0.016	1.47E-01	324405	3.36E-01
rs12617656	0.0511	0.0172	2.89E-03	315539	4.42E-02
rs2970933	-0.0312	0.016	5.14E-02	317703	1.99E-01
rs2287509	-0.0302	0.016	5.99E-02	313561	2.26E-01
**rs7565794**	**0.0487**	**0.0172**	**4.57E-03**	**314417**	**4.42E-02**
rs2909449	-0.0254	0.016	1.13E-01	314303	2.91E-01
rs2909450	-0.0194	0.0194	3.15E-01	312867	4.98E-01
**rs1014444**	**0.0457**	**0.0169**	**6.91E-03**	**314029**	**4.42E-02**
rs1014445	-0.0257	0.0161	1.10E-01	313491	2.91E-01
rs2268890	-0.0277	0.0161	8.42E-02	313207	2.75E-01
**rs2300757**	**0.0501**	**0.0172**	**3.53E-03**	**315305**	**4.42E-02**
rs12692646	0.0495	0.0172	4.00E-03	315377	4.42E-02
rs13409045	0.0459	0.0165	5.48E-03	313171	4.42E-02
rs2300755	0.0465	0.0165	4.87E-03	320741	4.42E-02
rs2268889	0.0527	0.0169	1.80E-03	322939	4.42E-02
rs4664443	0.0446	0.0164	6.46E-03	321675	4.42E-02
rs2284870	0.0484	0.0167	3.66E-03	322655	4.42E-02
rs6432708	-0.0215	0.0162	1.85E-01	304611	3.82E-01
rs10930040	0.0469	0.017	5.82E-03	323657	4.42E-02
rs71408196	0.0426	0.0271	1.15E-01	325475	2.91E-01
rs12995983	-0.0155	0.0181	3.93E-01	284981	5.19E-01
rs3788979	0.0398	0.0233	8.80E-02	324097	2.75E-01
**rs16822665**	**0.0468**	**0.0172**	**6.37E-03**	**321433**	**4.42E-02**
rs4664446	0.0296	0.0165	7.26E-02	265385	2.54E-01
rs873826	-0.0156	0.0163	3.39E-01	317019	4.98E-01
rs741529	0.0437	0.0255	8.67E-02	325877	2.75E-01
rs35280626	-0.0103	0.0172	5.48E-01	309315	6.49E-01
rs4436979	-0.0329	0.016	4.03E-02	319685	1.66E-01
rs2052352	0.0453	0.016	4.52E-03	321405	4.42E-02
rs13431489	0.0376	0.0162	2.01E-02	322201	9.53E-02
rs1990761	0.0232	0.0193	2.30E-01	158433	4.09E-01

Abbreviations: BETA, regression coefficient; SE, standard error; P-value for linear regression under the additive model; N, number of biobank participants; P.FDR, P-value of false discovery rate test; SNPs that also represent peaks of association with C-peptide AUC (0-120) in the PREVADIAB-2 cohort are highlighted in bold.

ESM Table 5 Quantitative trait locus analysis of plasma glucose levels at 0min, 30min and 120min during the OGTT with *DPP4* SNPs in NGT subjects.

			Glucose 0min			Glucose 30min			Glucose 120min	
**SNP**	**A**	**BETA**	**SE**	**P_{asymp}**	**BETA**	**SE**	**P_{asymp}**	**BETA**	**SE**	**$P_{asymp.}$**
rs10930036	T	0.24	0.48	6.19E-01	-2.28	1.53	1.37E-01	-1.26	1.19	2.92E-01
rs2052400	A	-0.72	0.46	1.18E-01	-4.47	1.45	2.18E-03	-3.42	1.13	2.58E-03
rs12617656	C	0.98	0.50	4.85E-02	4.93	1.56	1.65E-03	3.28	1.22	7.39E-03
rs2970933	A	-0.69	0.46	1.37E-01	-5.46	1.45	1.80E-04	-3.49	1.13	2.15E-03
rs2287509	G	-0.63	0.47	1.81E-01	-5.30	1.47	3.29E-04	-2.98	1.15	9.50E-03
rs7565794	C	0.89	0.49	7.23E-02	4.94	1.55	1.50E-03	3.36	1.21	5.66E-03
**rs2909449**	**G**	-0.75	0.46	**1.02E-01**	**-5.75**	**1.44**	**7.47E-05**	**-3.38**	**1.13**	**2.84E-03**
rs2909450	A	0.36	0.51	4.83E-01	3.55	1.62	2.82E-02	1.35	1.26	2.87E-01
rs1014444	G	0.85	0.48	7.72E-02	4.47	1.51	3.21E-03	3.23	1.18	6.38E-03
rs1014445	A	-0.53	0.47	2.56E-01	-5.07	1.46	5.59E-04	-3.37	1.14	3.28E-03
**rs2268890**	**A**	-0.68	0.46	**1.37E-01**	**-5.63**	**1.44**	**1.04E-04**	**-3.49**	**1.13**	**2.06E-03**
rs2300757	G	0.92	0.50	6.67E-02	4.93	1.57	1.80E-03	3.23	1.23	8.69E-03
rs12692646	A	0.97	0.50	5.29E-02	5.52	1.57	4.68E-04	2.67	1.24	3.13E-02
rs138687963	D	0.37	0.51	4.74E-01	3.74	1.62	2.09E-02	1.70	1.26	1.79E-01
rs13409045	T	0.22	0.46	6.26E-01	2.11	1.45	1.46E-01	1.80	1.13	1.11E-01
rs2300755	T	0.46	0.47	3.33E-01	3.03	1.49	4.14E-02	1.87	1.16	1.07E-01
rs2268889	A	0.71	0.49	1.53E-01	4.82	1.56	2.01E-03	2.39	1.22	5.04E-02
rs4664443	G	0.27	0.46	5.50E-01	2.36	1.45	1.03E-01	1.85	1.13	1.02E-01
rs2284870	G	0.51	0.48	2.91E-01	3.77	1.52	1.37E-02	2.44	1.19	4.08E-02
**rs6432708**	**C**	-0.58	0.47	**2.17E-01**	**-4.98**	**1.47**	**7.69E-04**	**-3.28**	**1.15**	**4.44E-03**
rs10930040	G	0.92	0.50	6.87E-02	5.01	1.59	1.65E-03	2.61	1.24	3.56E-02
rs71408196	I	1.12	0.72	1.22E-01	5.99	2.28	8.63E-03	3.13	1.78	7.92E-02
rs12995983	C	0.24	0.51	6.37E-01	-1.96	1.61	2.24E-01	-3.10	1.26	1.38E-02
rs3788979	A	0.53	0.76	4.91E-01	2.91	2.41	2.28E-01	0.68	1.88	7.16E-01
rs16822665	T	0.90	0.51	7.69E-02	5.17	1.59	1.20E-03	2.91	1.25	1.98E-02
**rs4664446**	**G**	-1.48	0.45	**1.10E-03**	**-6.10**	**1.42**	**1.89E-05**	**-2.62**	**1.12**	**1.91E-02**
rs873826	A	-0.33	0.48	4.98E-01	-2.67	1.53	8.14E-02	-2.08	1.19	8.05E-02
rs741529	A	-0.27	0.65	6.77E-01	4.22	2.05	3.98E-02	-0.84	1.60	5.99E-01
rs35280626	T	0.00	0.51	9.98E-01	-0.82	1.61	6.11E-01	-2.11	1.25	9.27E-02
rs4436979	C	-0.13	0.46	7.83E-01	-2.03	1.44	1.60E-01	-0.48	1.13	6.71E-01
rs2052352	T	-0.28	0.45	5.38E-01	0.47	1.41	7.37E-01	1.39	1.10	2.07E-01
rs13431489	G	-0.21	0.46	6.38E-01	1.27	1.44	3.78E-01	1.61	1.12	1.52E-01
rs1990761	G	-0.21	0.49	6.77E-01	-2.02	1.56	1.95E-01	-0.12	1.22	9.21E-01

Abbreviations: A, minor allele; BETA, regression coefficient; SE, standard error; P_{asymp}, asymptotic P-value for linear regression under the additive model adjusted for age and BMI; SNP's representing peaks of association are highlighted in bold.

ESM Table 6 Quantitative trait locus analysis of plasma C-peptide levels at 0min, 30min and 120min during the OGTT with *DPP4* SNPs in NGT subjects.

		**C-peptide 0min**			**C-peptide 30min**			**C-peptide 120min**		
**SNP**	**A**	**BETA**	**SE**	**P_{asymp}**	**BETA**	**SE**	**P_{asymp}**	**BETA**	**SE**	**$P_{asymp.}$**
rs10930036	T	-0.03	0.04	4.45E-01	-0.18	0.14	1.88E-01	-0.47	0.18	9.83E-03
rs2052400	A	-0.10	0.04	1.71E-02	-0.26	0.13	5.36E-02	-0.54	0.17	1.84E-03
rs12617656	C	0.12	0.04	5.94E-03	0.08	0.14	6.00E-01	0.64	0.19	7.07E-04
rs2970933	A	-0.10	0.04	1.43E-02	-0.27	0.13	4.40E-02	-0.56	0.17	1.45E-03
rs2287509	G	-0.10	0.04	1.70E-02	-0.29	0.14	3.01E-02	-0.53	0.18	2.47E-03
**rs7565794**	**C**	**0.11**	**0.04**	**1.02E-02**	**0.02**	**0.14**	**9.16E-01**	**0.66**	**0.19**	**4.04E-04**
rs2909449	G	-0.10	0.04	1.21E-02	-0.29	0.13	3.23E-02	-0.54	0.17	2.03E-03
rs2909450	A	0.00	0.05	9.18E-01	0.09	0.15	5.57E-01	0.02	0.19	9.03E-01
**rs1014444**	**G**	**0.12**	**0.04**	**4.25E-03**	**0.08**	**0.14**	**5.54E-01**	**0.66**	**0.18**	**2.86E-04**
rs1014445	A	-0.10	0.04	1.91E-02	-0.25	0.13	6.34E-02	-0.54	0.18	2.26E-03
rs2268890	A	-0.10	0.04	1.30E-02	-0.27	0.13	4.38E-02	-0.55	0.17	1.48E-03
**rs2300757**	**G**	**0.12**	**0.04**	**5.40E-03**	**0.03**	**0.15**	**8.26E-01**	**0.69**	**0.19**	**2.91E-04**
rs12692646	A	0.11	0.04	1.01E-02	0.07	0.15	6.30E-01	0.65	0.19	6.68E-04
rs138687963	D	-0.01	0.05	7.94E-01	0.07	0.15	6.41E-01	0.04	0.19	8.23E-01
rs13409045	T	0.09	0.04	2.08E-02	0.19	0.13	1.53E-01	0.42	0.17	1.50E-02
rs2300755	T	0.10	0.04	1.12E-02	0.22	0.14	9.96E-02	0.52	0.18	3.75E-03
rs2268889	A	0.11	0.04	1.08E-02	0.11	0.14	4.38E-01	0.56	0.19	2.80E-03
rs4664443	G	0.09	0.04	1.92E-02	0.20	0.13	1.37E-01	0.47	0.17	7.18E-03
rs2284870	G	0.12	0.04	3.63E-03	0.14	0.14	3.18E-01	0.57	0.18	1.92E-03
rs6432708	C	-0.11	0.04	8.85E-03	-0.28	0.14	4.29E-02	-0.54	0.18	2.44E-03
rs10930040	G	0.11	0.04	1.34E-02	0.05	0.15	7.29E-01	0.60	0.19	1.64E-03
rs71408196	I	0.15	0.06	2.16E-02	0.21	0.21	3.23E-01	0.79	0.27	4.09E-03
rs12995983	C	-0.09	0.04	3.80E-02	-0.21	0.15	1.50E-01	-0.49	0.19	1.08E-02
rs3788979	A	-0.04	0.07	5.04E-01	-0.07	0.22	7.57E-01	-0.10	0.29	7.19E-01
**rs16822665**	**T**	**0.12**	**0.04**	**7.71E-03**	**0.08**	**0.15**	**5.69E-01**	**0.65**	**0.19**	**7.03E-04**
rs4664446	G	-0.08	0.04	4.27E-02	-0.07	0.13	5.74E-01	-0.40	0.17	1.87E-02
rs873826	A	-0.09	0.04	4.39E-02	-0.07	0.14	5.93E-01	-0.40	0.18	3.06E-02
rs741529	A	0.04	0.06	4.45E-01	0.18	0.19	3.51E-01	0.33	0.25	1.82E-01
rs35280626	T	-0.06	0.04	2.16E-01	0.00	0.15	9.93E-01	-0.23	0.19	2.36E-01
rs4436979	C	0.02	0.04	5.60E-01	-0.08	0.13	5.56E-01	-0.15	0.17	3.81E-01
rs2052352	T	0.04	0.04	3.31E-01	0.16	0.13	2.02E-01	0.14	0.17	3.99E-01
rs13431489	G	0.02	0.04	5.99E-01	0.12	0.13	3.65E-01	0.17	0.17	3.16E-01
rs1990761	G	-0.02	0.04	6.32E-01	0.07	0.14	6.42E-01	-0.13	0.19	4.71E-01

Abbreviations: A, minor allele; BETA, regression coefficient; SE. standard error; P_{asymp}, asymptotic P-value for linear regression under the additive model adjusted for age and BMI; SNP's representing peaks of association are highlighted in bold.

ESM Fig. 1a

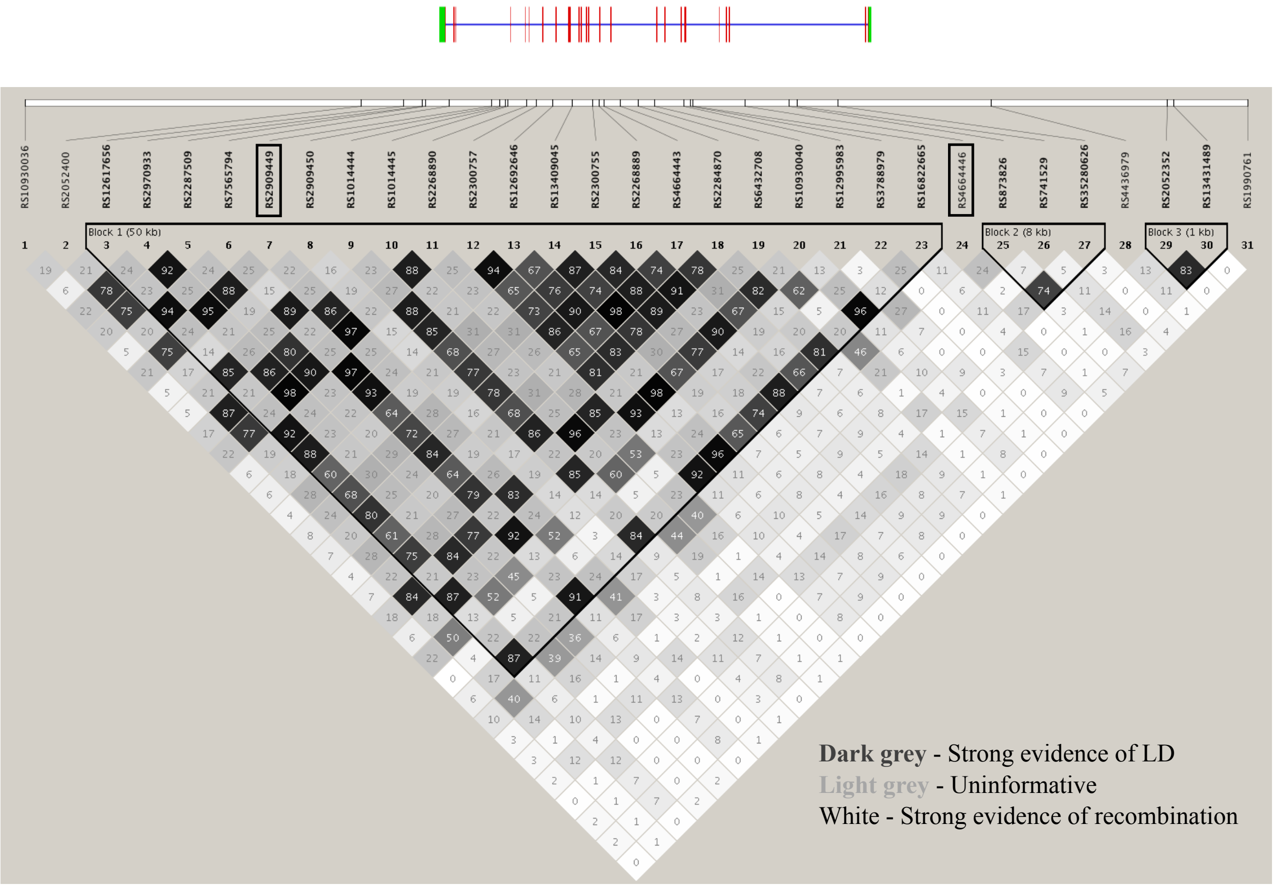

ESM Fig. 1b

Phenotype/ SNP		Covariates	
**Glucose (AUC)**	**rs2909449**	**rs2268890**	**rs6432708**
**rs4664446**	0.04	0.26	0.77
**C-peptide (0-120)**	**rs7565794**	**rs1014444**	**rs2300757**
**rs16822665**	0.41	0.09	0.79

ESM Fig. 1 (a) Linkage disequilibrium (LD) map of the genotyped *DPP4* SNPs with overimposed scaled representation of exon-intron structure of the *DPP4* gene (oriented from right to left). R-square values for pairwise LD were calculated using 969 subjects analyzed in this study. Color coding highlights a large LD block spanning from intron 3 to 23 (Block 1). Indels (rs138687963 and rs71408196) are excluded from the LD map. LD plot was generated by Haploview 4.2. software. (b) Analysis of strongest associated SNPs for glucose AUC and C-peptide AUC (0-120) conditioned to relevant associated SNPs. P-values of pairwise covariance analysis are represented.

ESM Figure 2

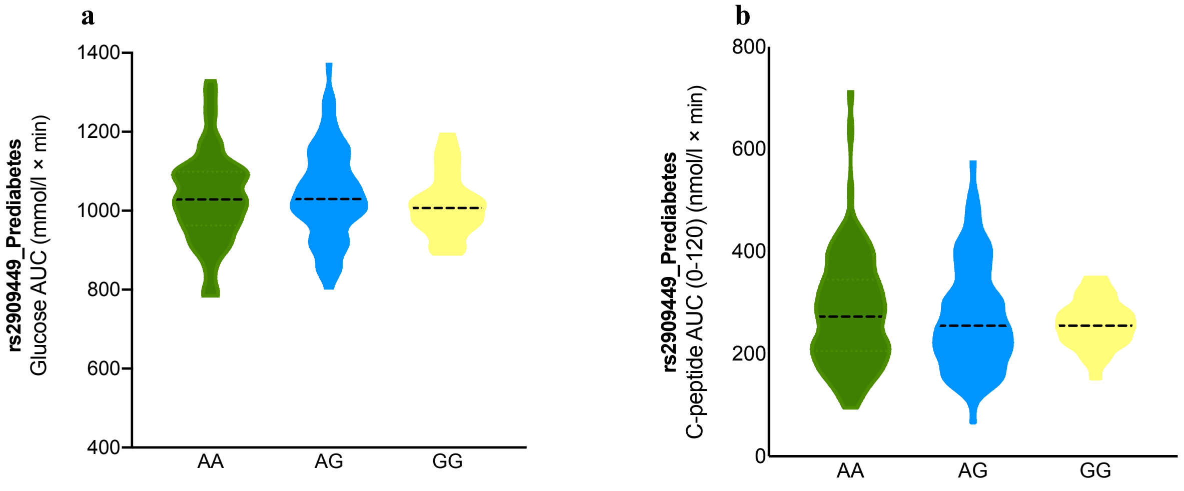

ESM Fig. 2 Violin plots of genotype-class effects of rs2909449 on plasma glucose AUC **(a)** and C-peptide AUC (0-120) **(b)** during OGTT (green, ancestral allele homozygotes; blue heterozygotes; yellow, minor allele homozygotes). Kruskal-Wallis test with Dunn's correction for multiple comparisons (Unpaired t-test Mann-Whitney). The plots represent the probability density, the median, the interquartile range and the 95% confidence interval of the phenotypic distributions per genotype class totaling 233 prediabetic participants.
